# Multicenter Hospital-Based Prospective Surveillance Study of Bacterial Agents Causing Meningitis and Seroprevalence of Different Serogroups of Neisseria meningitidis, Haemophilus influenzae Type b, and Streptococcus pneumoniae during 2015 to 2018 in Turkey

**DOI:** 10.1128/mSphere.00060-20

**Published:** 2020-03-25

**Authors:** Mehmet Ceyhan, Yasemin Ozsurekci, Sevgen Tanır Basaranoglu, Nezahat Gurler, Enes Sali, Melike Keser Emiroglu, Fatma Nur Oz, Nursen Belet, Murat Duman, Emel Ulusoy, Zafer Kurugol, Hasan Tezer, Aslinur Ozkaya Parlakay, Ener Cagri Dinleyici, Umit Celik, Solmaz Celebi, Ahmet Faik Oner, Mehmet Ali Solmaz, Adem Karbuz, Nevin Hatipoglu, Ilker Devrim, Ilknur Caglar, Sefika Elmas Bozdemir, Emine Kocabas, Ozlem Ozgur Gundeslioglu, Murat Sutcu, Ozge Metin Akcan, Necdet Kuyucu, Fesih Aktar, Soner Sertan Kara, Havva Ozlem Altay Akisoglu, Nilden Tuygun, Zeynep Diyar Tamburaci Uslu, Eda Karadag Oncel, Cihangul Bayhan, Ali Bulent Cengiz

**Affiliations:** a Department of Pediatric Infectious Diseases, Hacettepe University Faculty of Medicine, Ankara, Turkey; b Faculty of Medicine, Department of Medical Microbiology, Istanbul University, Istanbul, Turkey; c Department of Pediatric Infectious Diseases, Sanliurfa Education and Training Hospital, Sanliurfa, Turkey; d Department of Pediatric Infectious Diseases, Selcuk University, Konya, Turkey; e Department of Pediatric Infectious Diseases, Sami Ulus Maternity and Children’s Research and Training Hospital, Ankara, Turkey; f Department of Pediatric Infectious Diseases, Faculty of Medicine, Dokuz Eylul University, Izmir, Turkey; g Department of Pediatric Emergency, Faculty of Medicine, Dokuz Eylul University, Izmir, Turkey; h Department of Emergency, Dr. Behcet Uz Children’s Hospital, Izmir, Turkey; i Department of Pediatric Infectious Diseases, Ege University Faculty of Medicine, Izmir, Turkey; j Department of Pediatric Infectious Diseases, Gazi University Faculty of Medicine, Ankara, Turkey; k Department of Pediatric Infectious Diseases, Diskapi Yildirim Beyazit Training and Research Hospital, Ankara, Turkey; l Department of Pediatrics, Faculty of Medicine, Eskisehir Osmangazi University, Eskisehir, Turkey; m Department of Pediatric Infectious Diseases, Adana Numune Training and Research Hospital, Adana, Turkey; n Department of Pediatric Infectious Diseases, Faculty of Medicine, Bursa Uludag University, Bursa, Turkey; o Department of Pediatric Infectious Diseases, Faculty of Medicine, Van Yuzuncu Yıl University, Van, Turkey; p Department of Pediatrics, Faculty of Medicine, Van Yuzuncu Yıl University, Van, Turkey; q Department of Pediatric Infectious Diseases, Okmeydanı Training and Research Hospital, Istanbul, Turkey; r Department of Pediatric Infectious Diseases, Bakirkoy Dr Sadi Konuk Training and Research Hospital, Istanbul, Turkey; s Department of Pediatric Infectious Diseases, Dr. Behcet Uz Children’s Hospital, Izmir, Turkey; t Department of Pediatric Infectious Diseases, Kayseri Education and Research Hospital, Kayseri, Turkey; u Department of Pediatric Infectious Diseases, Cukurova University Faculty of Medicine, Adana, Turkey; v Department of Pediatric Infectious Diseases, Konya Training and Research Hospital, Konya, Turkey; w Department of Pediatric Infectious Diseases, Faculty of Medicine, Mersin University, Mersin, Turkey; x Department of Pediatrics, Faculty of Medicine, Dicle University, Diyarbakir, Turkey; y Department of Pediatric Infectious Diseases, Erzurum Regional Training and Research Hospital, Erzurum, Turkey; z Department of Microbiology, Sami Ulus Maternity and Children’s Research and Training Hospital, Ankara, Turkey; aa Department of Pediatric Emergency, Sami Ulus Maternity and Children’s Research and Training Hospital, Ankara, Turkey; bb Department of Pediatric Cardiology, Akdeniz University, Antalya, Turkey; cc Department of Pediatric Infectious Diseases, Izmir University of Health Sciences Tepecik Training and Research Hospital, Izmir, Turkey; University of Nebraska Medical Center

**Keywords:** meningitis, Turkey, *N. meningitidis*, *S. pneumoniae*, Hib, epidemiology, surveillance

## Abstract

The etiology of bacterial meningitis in Turkey changed after the implementation of conjugated vaccines against Streptococcus pneumoniae and Haemophilus influenzae type b (Hib) in the Turkish National Immunization Program (NIP). Administration of Hib vaccine and PCV-7 (7-valent pneumococcal conjugate vaccine) was implemented in NIP in 2006 and 2009, respectively. In 2011, PCV-7 was replaced with PCV-13. Meningococcal vaccines have not yet been included in Turkish NIP. This prospective study comprised 27 hospitals located in seven regions of Turkey and represented 45% of the population. Children aged between 1 month and 18 years who were hospitalized with suspected meningitis were included. Cerebrospinal fluid (CSF) samples were collected, and bacterial identification was made according to the multiplex PCR assay results. During the study period, 994 children were hospitalized for suspected meningitis, and Hib (*n* = 3, 2.4%), S. pneumoniae (*n* = 33, 26.4%), and Neisseria meningitidis (*n* = 89, 71%) were detected in 125 samples. The most common meningococcal serogroup was MenB. Serogroup W comprised 13.9% (*n* = 5) and 7.5% (*n* = 4) of the meningococci in 2015 to 2016 and 2017 to 2018, respectively. Serogroup C was not detected. There were four deaths in the study; one was a pneumococcus case, and the others were serogroup B meningococcus cases. The epidemiology of meningococcal diseases has varied over time in Turkey. Differing from the previous surveillance periods, MenB was the most common serogroup in the 2015-to-2018 period. Meningococcal epidemiology is so dynamic that, for vaccination policies, close monitoring is crucial.

**IMPORTANCE** Acute bacterial meningitis (ABM) is one of the most common life-threatening infections in children. The incidence and prevalence of ABM vary both geographically and temporally; therefore, surveillance systems are necessary to determine the accurate burden of ABM. The Turkish Meningitis Surveillance Group has been performing a hospital-based meningitis surveillance study since 2005 across several regions in Turkey. Meningococcus was the major ABM-causing agent during the 2015-to-2018 period, during which MenB was the dominant serogroup.

## Abstract (precis)

Acute bacterial meningitis (ABM) is one of the most common life-threatening infections in children. The incidence and prevalence of ABM vary both geographically and temporally; therefore, surveillance systems are necessary to determine the accurate burden of ABM. The Turkish Meningitis Surveillance Group has been performing a hospital-based meningitis surveillance study since 2005 across several regions in Turkey. Meningococcus was the major ABM-causing agent during the 2015-to-2018 period, during which MenB was the dominant serogroup.

## INTRODUCTION

Acute bacterial meningitis (ABM) is one of the most common life-threatening infections in infants and children (1). While endemic bacterial meningitis is a relatively less frequent illness in developed countries, the probability of occurrence of endemic and epidemic bacterial meningitis in undeveloped countries remains a major concern (2). Given that the incidence and prevalence of ABM continually vary both geographically and temporally, while surveillance systems differ between countries, accurate information on the burden of ABM remains unavailable. The epidemiology of the most frequent causes of ABM, Neisseria meningitidis, Streptococcus pneumoniae, and Haemophilus influenzae type b (Hib), may change considerably according to the geographic region and immunization schedules. Genomic alterations, especially of meningococci, may increase the epidemic potential of agents (3–6).

The prevalence of Hib-associated and S. pneumoniae-associated meningitis has widely decreased in many regions worldwide due to the success of vaccination (7). For children under 1 year of age, the Turkish National Immunization Program (NIP) started Hib vaccination in 2006 and 7-valent pneumococcal conjugate vaccine (PCV-7) administration in 2009. PCV-7 was replaced by PCV-13 in November 2011, with a 3 + 1 schedule. The PCV-13 schedule was changed to 2 + 1 in March 2019. In the most recent report of World Health Organization (8), the percentage of children vaccinated with three doses of Hib was 98%. The percentage of children who were vaccinated with three doses of PCV was 97%.

There are vaccines available for all major disease-causing serogroups of N. meningitidis (A, B, C, W, and Y) except serogroup X, which has multiple versions in development (9). Still, it should be noted that the vaccine targeting serogroup B is only broadly protective and does not cover all strains causing the disease (10). Meningococcal vaccines are serogroup specific or, in the case of the vaccines targeting serogroup B, protein-specific (11). Except for high-risk groups, meningococcal vaccines are not used in routine childhood immunization programs in Turkey, and they have not yet been implemented in the NIP.

Since the distributions of these meningitis-causing pathogens, as well as of their serogroups, differ widely by geographic region and display change in time, the Turkish Meningitis Surveillance Group has been performing a hospital-based meningitis surveillance study since 2005 across several regions in Turkey.

## RESULTS

A total of 994 suspected meningitis cases with cerebrospinal fluid (CSF) samples were studied between 2015 and 2018. The median age of the cases was 3.5 years (interquartile range [IQR], 0.83 to 9.0), with a male-to-female ratio of 3:2. Overall, 125 cases (12.5%) had a confirmed meningitis, among which 89 (71%) were N. meningitidis, 33 (26.4%) were S. pneumoniae, and 3 (2.4%) were Hib. The number of culture-confirmed meningitis cases was 23 (18.4%), where 16 had meningococcal and 7 had pneumococcal meningitis. The rest of the confirmed cases (*n* = 102, 81.6%) were diagnosed solely by PCR. All of the culture-positive cases were also PCR positive. Distributions of microorganisms according to the 2-year periods of 2015 to 2016 and 2017 to 2018 are detailed in Table 1. In both periods, among the identified meningococci, the most common serogroup was MenB (*n* = 16, 44.4% in 2015 to 2016; *n* = 29, 54.7% in 2017 to 2018). Serogroup W constituted 13.9% (*n* = 5) and 7.5% (*n* = 4) of the meningococci in 2015 to 2016 and 2017 to 2018, respectively. In 33% of the cases in 2015 to 2016 and 30% of the cases in 2017 to 2018, the serogroups could not be determined. No MenC was detected throughout the surveillance period. Among the patients with confirmed meningitis cases, four died; one was a pneumococcus patient, and three were MenB patients, with a case-fatality rate of 3.3% for all N. meningitidis cases.

**TABLE 1:** Distribution of causative agents of bacterial meningitis and meningococcal serogroups during 2015 to 2018 in Turkey

Causative bacterial species or serogroup	Value for samples collected during surveillance period:			
	2015–2016		2017–2018	
	*n*	%	*n*	%
Neisseria meningitidis	36	73.5	53	69.7
Serogroup W	5	13.9	4	7.5
Serogroup B	16	44.4	29	54.7
Serogroup A	1	2.8	2	3.8
Serogroup C	0	0	0	0
Serogroup Y	1	2.8	1	1.9
Serogroup X	1	2.8	1	1.9
Nongroupable	12	33.3	16	30.2
Streptococcus pneumoniae	12	24.5	21	27.6
Haemophilus influenzae type b	1	2	2	2.6

Total	49	100	76	100

### Annual incidence and age distribution.

The annual incidence of laboratory-confirmed ABM in children was calculated per 100,000 population. It was 0.19 in the 2015–2016 period and 0.29 in 2017–2018 period. For pneumococcal meningitis, the incidence was 0.1 in the 2015–2016 period and 0.18 in the 2017–2018 period. For meningococcal meningitis, the incidence was 0.3 in the 2015–2016 period and 0.4 in the 2017–2018 period, respectively. Among the meningococcal cases, 34.8% of the children were ≤1 year of age, 27% were 1 to 4 years of age, 21.3% were 5 to 9 years of age, 13.4% were 10 to 14 years of age, and 3.3% were 15 to 18 years of age. Among the pneumococcal cases under 15 years of age, the age distribution was 21%, 33.3%, 33.3%, and 12%, respectively (there were no pneumococcal meningitis cases older than 15 years). One Hib case was younger than 1 year of age, and 2 were in the age group of 1 to 4 years.

### Surveillance according to previous periods.

**FIG 1:**
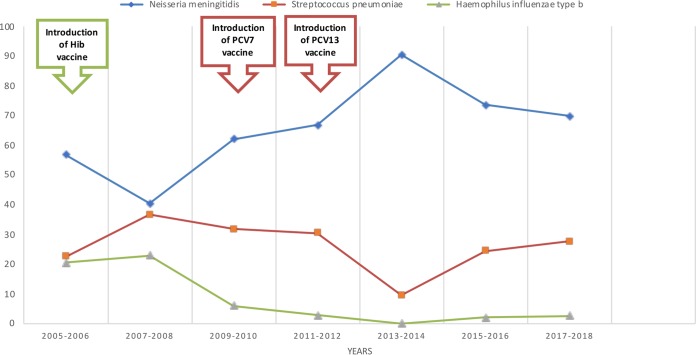
Percent distribution of causative agents of bacterial meningitis in Turkey according to years.

The Turkish Meningitis Surveillance Team has been following the pediatric ABM cases since 2005 (12–14). Data representing results of the follow-up of etiological agents of meningitis cases from 2005 to 2018 are shown in Fig. 1. Throughout the surveillance, the leading cause of meningitis was N. meningitidis in all periods. After introduction of the Hib vaccine, Hib-associated meningitis cases dropped down from 20.6% (2005 to 2006) to 2.4% (2015 to 2018). Before the introduction of the PCV7 vaccine, the frequency of pneumococcal meningitis was 62%, while it declined to 9.6% in the 2013–2014 period. In the present study, the pneumococcal meningitis percentages were 24.5% and 27.6% in the 2015–2016 and 2017–2018 periods, respectively.

**FIG 2:**
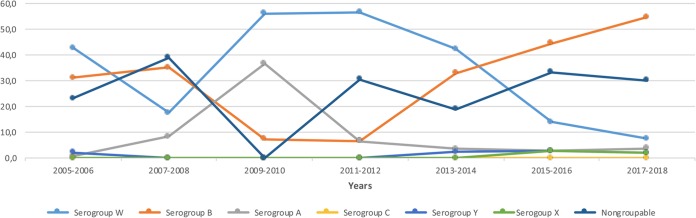
Percent distribution of N. meningitidis serogroups in Turkey according to years.

Data representing results from the surveillance of pediatric meningococcal meningitis cases from 2005 to 2018 in Turkey are shown in Fig. 2. Serogroup B was detected in 31.2%, 6.5%, and 54.7% of cases in the 2005–2006, 2011–2012, and 2017–2018 periods, respectively. The proportion of serogroup W cases was 56.5% in 2011 to 2012, while the frequency declined to 7.5% in the 2017–2018 period. From the start of surveillance to the 2017–2018 period, serogroup C type N. meningitidis was not detected.

## DISCUSSION

Observing the burden of ABM, defining the causative agents, and demonstrating the changes in epidemiology are critical points in establishing effective strategies for prevention of the disease. In the present surveillance study, the leading cause of ABM in children between 1 month and 18 years of age was found to be N. meningitidis, with MenB as the most frequent serogroup in 2015 to 2018, with a decrease in the total number of meningitis cases. This contrasts with our previous findings, in which MenW was the leading cause of meningococcus-associated bacterial meningitis. From 2007 to 2012, the prevalence of MenW increased and reached 56.5% of meningococcal meningitis cases (13). In the 2013–2014 study, MenW was the leading agent and constituted 42.4% (14); however, the prevalence of MenB started increasing, and it reached 54.7% in 2017 to 2018. The prevalence of serogroups A and X did not change much during the surveillance starting from 2005 and stayed lower than 10%. Before the present data were determined, the relative overall prevalences calculated for different capsular groups of meningococcal diseases in Turkey had been similar to those in Saudi Arabia, with MenA and MenW being the most common capsular groups detected (15). Moreover, the nasopharyngeal carriage study of meningococci in adolescents and young adults showed that the most prevalent serogroup at the beginning of 2015 was MenC, and a history of Hajj/Umrah travel in the last year for householders was significantly related to nasopharyngeal carriage (16). However, the present data display the prevalence propensities of our country becoming similar to Europe, the Americas, and the Western Pacific (17). In addition, the present case-fatality rate for N. meningitidis was higher than that in previous periods, rendering routine meningococcal vaccination necessary for our population. Conjugated meningococcal ACWY vaccines have been available in Turkey for approximately 8 years, but not in the NIP. In addition, Hajj and Umrah pilgrims started to be vaccinated with the conjugated meningococcal ACWY vaccines from the beginning of 2018. Therefore, both the reduced case numbers and serogroup shift from MenW to MenB may possibly be explained by the use of those vaccines for many years. Since November 2018, there has been a 4CMenB vaccine available in Turkey; this has the potential to decrease meningitis cases caused by MenB as well in our further epidemiological survey.

Following the meningococcal proportion, S. pneumoniae constituted 26.4% of the cases. Before implementation of the Hib and pneumococcal conjugate vaccines in 2006 and 2009, respectively, in the Turkish NIP, these agents comprised more than 40% of the pediatric ABM cases (13). The Hib vaccine resulted in a significant decrease in Hib-associated ABM over time, and the percentage was as low as 2% in the present study period. As a consequence of the implementation of PCV7 in 2009 and the switch to PCV13 in 2011, the proportion of pneumococcal meningitis cases decreased from 31.8% in 2009 to 2010 (13) to 9.6% in 2013 to 2014 (14). The annual incidence of pneumococcal meningitis was 0.7/100,000 at the beginning of surveillance in the 2005–2006 period (13) and decreased to 0.08 in 2014. In the 2017–2018 period, the incidence of pneumococcal meningitis was 0.18 per 100,000, representing an incremental increase. Many studies have demonstrated serotype (ST) replacement with the wide use of PCV7 and PCV13, decreasing the incidence of vaccine-type invasive pneumococcal disease in the population (18–20). Findings of serotype replacement disease after the introduction of PCV from several surveillance programs around the world suggested that serotype replacement would not be expected to occur within 2 years after the introduction of PCV13 (21, 22). The incremental changes in the rates of pneumococcal meningitis cases in 2017 to 2018 found in the present study may have been due to serotype replacement as a result of PCV13 use in a 3 + 1 schedule in Turkish NIP up to January 2019, when the schedule was changed to 2 + 1.

Although Turkey has hosted a large number of Syrian immigrants in the last 5 years due to Syrian civil war, the ABM incidence did not increase during the present study period. The annual ABM incidence was 3.5 in the 2005–2006 period (13) and was 0.9 cases/100,000 in 2014 (14). Further, it decreased to 0.19 in the 2015–2016 period and to 0.29 in the 2017–2018 period. We did not observe any increase that can be associated with the change of population characteristics. The satisfying results may be associated with the massive vaccination efforts exerted by health authorities at the border and throughout the country for this population. The present surveillance did not include cases from the refugee population because of the legal policy regulations during the study period. Therefore, the real influence of the refugee population on the meningitis epidemiology is not clear.

While nongroupable meningococci are commonly regarded as less life-threatening disease-causing serogroups compared with encapsulated ones, cases with invasive meningococcal disease caused by nongroupable serogroups vary in severity and may be fatal (23, 24). Meningococcal disease with nongroupable groups has been reported in immunocompetent patients (25). McNamara et al. (26) demonstrated that the nongroupable cases had a case fatality rate, presentation, and risk of sequelae comparable to those for meningococcal disease caused by serogroupable strains In our previous surveillance reports, nongroupable meningococci consisted of 26.4% and 18.8% of cases in the 2005–2012 and 2013–2014 periods, respectively. In the present study, among the meningococcal cases, over 30% were in the nongroupable group. Although there are no exact data for meningococcal immunization rates in Turkey, this relative increase may be related to the decline in the incidence of infections by capsulated serogroups resulting from the use of the ACWY vaccine in high-risk patients and in general practice for children under 2 years of age.

There were several limitations in the present study. First, we do not know the molecular details of our isolates and thus cannot predict disease severity. There have been reports of detection of highly invasive ST clones all over the world with a high case-fatality ratio (27, 28). Second, there have been some meningitis cases caused by MenC, particularly in the Northwest part of Turkey; however, there were no cases caused by MenC in the present study. Third, we were not able to acquire data representing the immunization status of the study population. Despite the mentioned limitations, we believe that our surveillance data highlight the epidemiologic trends of meningitis etiology in addition to the influence on vaccine policy development in Turkey over the years examined.

In conclusion, meningococcal seroepidemiology may change over the years as has occurred in Turkey, where MenB was the dominant causative agent of IMD cases in recent years. To make decisions about vaccination policy, especially in countries where meningococcal vaccines were not implemented in the NIP, including Turkey, ongoing surveillance is definitely needed.

## MATERIALS AND METHODS

### Surveillance system and patient enrollment.

A hospital-based, prospective, multicenter study was carried out in Turkey between January 2015 and December 2018. Twenty-seven hospitals located in seven different geographical regions of Turkey were included. The surveyed population comprised 45% of the population in Turkey. The study was approved by Zekai Tahir Burak Women’s Health Research and Training Hospital University Ethics Commission (approval numbers 16/2013 and 35/2015). Patients between 1 month and 18 years of age with suspected ABM were enrolled in the study according to the following standard criteria: any sign of meningitis (fever [≥38°C], vomiting [≥3 episodes in 24 h], headache, signs of meningeal irritation [bulging fontanel, Kernig or Brudzinski sign, or neck stiffness]) in children >1 year of age; fever without any known origin; impaired consciousness (Blantyre coma scale value of <4 if younger than 9 months of age and <5 if older than 9 months of age) (29); total weakness (inability to sit unassisted if ≥9 months of age and inability to breastfeed if <9 months) in those younger than 1 year of age; and seizures (other than those regarded as simple febrile seizures, with full recovery within 1 h). After written parental consent, cerebrospinal fluid (CSF) was collected. In addition, data representing demographic characteristics, clinical findings, underlying diseases, prior antibiotic use, treatment, laboratory evaluation, and outcome were obtained.

### Laboratory methods.

In local hospitals, blood specimens and CSF specimens were cultured immediately after collection, and CSF leukocyte counts were noted. Biochemical studies of CSF samples, such as measurement of protein and glucose levels, were performed. Analyses were performed and patient condition assessed according to the following criteria: (i) >10 leukocytes/mm^3^ in the CSF; (ii) higher CSF protein levels than normal for the patient’s age; (iii) lower CSF glucose levels than normal for the patient’s age. If these tests were positive, culture, PCR, Gram stain, or antigen detection tests were conducted. A confirmed bacterial meningitis diagnosis was defined as laboratory confirmation of meningitis by culture positivity in blood/CSF and/or PCR positivity in CSF or identification of the presence of meningococci, pneumococci, and Hib (with Gram stain or antigen detection) in CSF. Confirmed bacterial meningitis caused by other bacteria was not included in the study. CSF samples (minimum of 0.5 ml) were stored at −20°C until transportation to the Central Laboratory at the Hacettepe University Medical School Department of Pediatric Infectious Diseases, Ankara, Turkey, for PCR analysis. Convenient bacterial isolates were also conveyed to the Central Laboratory and recultured on chocolate and blood agars and grown at 37°C in 5% CO_2_. Suspected meningococcal colonies were determined by Gram staining, an oxidase test, and an Api rapid carbohydrate utilization test (API NH Ref. 10400; bioMérieux, Germany). Antigenic formula-based (serogroup: serotype: serosubtype) phenotypic identification of meningococcal isolates was performed using the standard methods in the Meningococcal Reference Unit, Health Protection Agency, Manchester, United Kingdom (30–33). For multiplex PCR studies, all the transferred samples were stored at −80°C in the Central Laboratory and melted immediately before each test. DNA was isolated according to methods used in previous surveillance studies (12–14). A single-tube multiplex PCR assay was performed for the simultaneous identification of bacterial agents. The specific gene targets were *ctrA*, *bex*, and *ply* for N. meningitidis, Hib, and S. pneumoniae, respectively (12, 30). PCR was performed by using a DNA thermal cycler (GeneAmp PCR system model 9700; Applied Biosystems, Foster City, CA, USA). The samples in which positivity for N. meningitidis was detected were further serogrouped (into serogroups A, B, C, W, Y, and X) on the basis of the presence of oligonucleotides in the *ctrA* X gene for serogroup X; in the *siaD* gene for serogroups B, C, W, and Y; and in orf-2 of a gene cassette required for serogroup A (30, 34). All the amplicons were analyzed by electrophoresis on standard 3% agarose gels and visualized using UV fluorescence. A negative control consisting of distilled water and a positive control consisting of an appropriate reference strain (S. pneumoniae ATCC 49613; Hib ATCC 10211; N. meningitidis serogroup C L94 5016, serogroup A M99 243594, serogroup Y M05 240122, serogroup W M05 240125, serogroup B M05 240120, or serogroup X M405 ATCC 35560) were analyzed. The primers for each microorganism are shown in Table 2.

**TABLE 2:** Oligonucleotides and primers for *Haemophilus influenzae*, *Streptococcus pneumoniae*, and *Neisseria meningitidis* and for serogroups of meningococci

Oligonucleotide	Microorganism	Sequence (5′–3′)
ctrA F	Neisseria meningitidis	GCT GCG GTA GGT GGT TCA A
ctrA R	Neisseria meningitidis	TTG TCG CGG ATT TGC AAC TA
bex F	Haemophilus influenzae	TAT CAC ACA AAT AGC GGT TGG
bex R	Haemophilus influenzae	GGC CAA GAG ATA CTC ATA GAA CGT T
ply F	Streptococcus pneumoniae	TGC AGA GCG TCC TTT GGT CTA T
ply R	Streptococcus pneumoniae	CTC TTA CTC GTG GTT TCC AAC TTG A
orf 2 F	Neisseria meningitidis serogroup A	CGC AAT AGG TGT ATA TAT TCT TCC
orf 2 R	Neisseria meningitidis serogroup A	CGT AAT AGT TTC GTA TGC CTT CTT
siaD B F	Neisseria meningitidis serogroup B	GGA TCA TTT CAG TGT TTT CCA CCA
siaD B R	Neisseria meningitidis serogroup B	GCA TGC TGG AGG AAT AAG CAT TAA
siaD C F	Neisseria meningitidis serogroup C	TCA AAT GAG TTT GCG AAT AGA AGG T
siaD C R	Neisseria meningitidis serogroup C	CAA TCA CGA TTT GCC CAA TTG AC
siaD W F	Neisseria meningitidis serogroup W	CAG AAA GTG AGG GAT TTC CAT A
siaD W R	Neisseria meningitidis serogroup W	CAC AAC CAT TTT CAT TAT AGT TAC TGT
siaD Y F	Neisseria meningitidis serogroup Y	CTC AAA GCG AAG GCT TTG GTT A
siaD Y R	Neisseria meningitidis serogroup Y	CTG AAG CGT TTT CAT TAT AAT TGC TAA
ctrA X F	Neisseria meningitidis serogroup X	ATG TCA ACC ATG GCG C
ctrA X R	Neisseria meningitidis serogroup X	TAA TTT AGT TCT ACC C

### Statistical analysis.

Statistical analyses were performed using the commercial package SPSS for Windows version 19.0 (SPSS, Inc., Chicago, IL, USA). Values for numerical variables were provided as means and standard deviations or medians (interquartile ranges [IQRs]), depending on the normality of distribution. Categorical variables were given as numbers and total percentages.
